# Generation of a Highly Reactive Chicken-Derived Single-Chain Variable Fragment against *Fusarium verticillioides* by Phage Display

**DOI:** 10.3390/ijms13067038

**Published:** 2012-06-07

**Authors:** Zu-Quan Hu, Jin-Long Liu, He-Ping Li, Shu Xing, Sheng Xue, Jing-Bo Zhang, Jian-Hua Wang, Greta Nölke, Yu-Cai Liao

**Affiliations:** 1 Molecular Biotechnology Laboratory of Triticeae Crops, Huazhong Agricultural University, Wuhan 430070, China; E-Mails: huzuquan@webmail.hzau.edu.cn (Z.-Q.H.); liujinlong@webmail.hzau.edu.cn (J.-L.L.); hepingli@mail.hzau.edu.cn (H.-P.L.); xingshu2001@webmail.hzau.edu.cn (S.X.); shengxue@webmail.hzau.edu.cn (S.X.); jingbozhang@mail.hzau.edu.cn (J.-B.Z.); jianhuawang@webmail.hzau.edu.cn (J.-H.W.); 2 College of Plant Science and Technology, Huazhong Agricultural University, Wuhan 430070, China; 3 College of Life Science and Technology, Huazhong Agricultural University, Wuhan 430070, China; 4 Department Plant Biotechnology, Fraunhofer IME, Aachen 52074, Germany; E-Mail: greta.noelke@ime.fraunhofer.de; 5 National Center of Plant Gene Research (Wuhan), Wuhan 430070, China

**Keywords:** chicken antibody, enzyme-linked immunosorbent assays (ELISAs), *Fusarium verticillioides*, single-chain variable fragment (scFv), phage display, immunofluorescence labeling

## Abstract

*Fusarium verticillioides* is the primary causal agent of Fusarium ear and kernel rot in maize, producing fumonisin mycotoxins that are toxic to humans and domestic animals. Rapid detection and monitoring of fumonisin-producing fungi are pivotally important for the prevention of mycotoxins from entering into food/feed products. Chicken-derived single-chain variable fragments (scFvs) against cell wall-bound proteins from *F. verticillioides* were isolated from an immunocompetent phage display library. Comparative phage enzyme-linked immunosorbant assays (ELISAs) and sequencing analyses identified four different scFv antibodies with high sensitivity. Soluble antibody ELISAs identified two highly sensitive scFv antibodies, FvCA3 and FvCA4, with the latter being slightly more sensitive. Three-dimensional modeling revealed that the FvCA4 may hold a better overall structure with CDRH3, CDRL1 and CDRL3 centered in the core region of antibody surface compared with that of other scFvs. Immunofluorescence labeling revealed that the binding of FvCA4 antibody was localized to the cell walls of conidiospores and hyphae of *F. verticillioides*, confirming the specificity of this antibody for a surface target. This scFv antibody was able to detect the fungal mycelium as low as 10^−2^ μg/mL and contaminating mycelium at a quantity of 10^−2^ mg/g maize. This is the first report that scFv antibodies derived from phage display have a wide application for rapid and accurate detection and monitoring of fumonisin-producing pathogens in agricultural samples.

## 1. Introduction

*Fusarium verticillioides* is one of the most important fungal pathogens causing epidemic diseases through maize and many cereal crops worldwide. This species is the primary causal agent of maize ear and kernel rot, and infection takes place both in the field and during storage. Various mycotoxins, including fumonisins, moniliformin, fusarin C and fusarinic acid are produced during the infection, and directly accumulate in grains, thus, entering into food/feed chains [1–3]. These mycotoxins are known to be toxic to humans and domestic animals, causing serious diseases such as equine leukoencephalomalacia (ELEM), porcine pulmonary edema (PPE) and cancer in animals [3]. The high incidence of fumonisin B_1_ in maize and maize-based products has been associated with disruption of sphingolipid biosynthesis [4,5], hepato- and nephrotoxicity [6,7], and immuno-suppressive effect in humans [8,9]. Moreover, the frequent interaction of fumonisins with aflatoxins increases the toxicity of the latter [10,11] and fumonisins potentially provoke the occurrence of esophageal cancer [12–14] and liver cancer [14,15] in humans. The International Agency for Research on Cancer (IARC) has suggested that fumonisin B_1_ could possibly be carcinogenic to humans and therefore classified fumonisin B_1_ as class 2B. Thus, investigation of antibodies reactive to *F. verticillioides* is pivotally important for the efficient prevention and control of fumonisin mycotoxins in food and feedstuff products.

Maize is the largest crop in China in terms of acreage and productivity [16]. The frequent incidence of ear and kernel rot in maize caused by *F. verticillioides* and high doses of fumonisin contamination have been reported [14,15,17,18], as eco-environments and crop rotation systems in the maize growing regions are favorable for *F. verticillioides* infection [19–21]. Therefore, efficient detection and monitoring of fumonisin-producing fungi are essential to prevent mycotoxin contamination in food/feed products. However, rapid and accurate detection of toxigenic *F. verticillioides* strains is a challenge due to labor- and cost-extensive procedures through biological or molecular approaches, which requires culture of fungi *in vitro* and subsequent morphological and molecular identification. Furthermore, all these methods need expertise and facilities, thus are difficult to be performed in remote regions or country sides. Enzyme-linked immunosorbent assay (ELISA) is a simple procedure and does not require expertise and facilities. To develop an ELISA for detection of *F. verticillioides*, generations of highly sensitive antibodies against the surface antigens of these fungal pathogens is required.

Different types of antibodies, such as polyclonal antisera [22–25], monoclonal antibodies (mAbs) [26–29] and single-chain variable fragments (scFvs) [30–32] specific to various antigens of fungal pathogens have been generated. These antibodies were not only used for detection of pathogens [22–26,28–31] but also for the inhibition of fungal growth [27] and enhancement of resistance in plants [32–36]. To date, only polyclonal antibodies have been developed for immunological detection of *F. verticillioides* [24,25]. ScFv antibodies apparently have some advantages over polyclonal antisera or mAbs and can be isolated together with their coding sequences by phage display [37]. These antibodies carry only variable fragments and their relatively small size allows for easy genetic manipulation and molecular evolution to further improve their affinity [38–40]. In addition, fungus-specific scFvs, either alone or as a fusion with other molecules, have been shown to confer resistance to pathogens in transgenic plants [32–34].

A chicken-derived recombinant antibody is a preferable choice, since it is technically easier to generate than other animal species and displays high specificity and affinity [41–44]. Chickens preserve their diversity by gene rearrangement and recombination even though they possess only one functional V segment and one functional J segment in the immunoglobulin heavy and light chain loci [45–47]. The peculiar mechanism of immunoglobulin gene diversification leads to only one set of primers required for each antibody chain, instead of the mixtures needed for amplification of variable gene families from other animals, although previous studies have indicated that chickens produced lower antisera avidity than other animals such as rabbit and sheep [48,49].

In the present study, phage display was used to isolate high affinity chicken-derived scFv antibodies against *F. verticillioides*. A phagemid library was constructed with cDNA transcribed from splenic mRNA of chickens immunized with cell wall-bound proteins (CWPs) isolated from a representative *F. verticillioides* strain from maize rot in China. After several rounds of panning by phage display, four different scFv antibodies reactive to CWPs were isolated. Comparative analyses of their binding capacity and three-dimensional structures revealed that two antibodies display a high affinity and proper structures in their complementarity determining regions (CDRs). Immunofluorescence labeling localized a specific binding to the surface of mycelia and conidiospores. The best scFv antibody, FvCA4, was able to detect mycelium as low as ~10^−2^ μg/mL in PBS or ~10^−2^ mg/g (*i.e.*, 0.1 μg/mL) of mycelium in maize grains, suggesting a promising application for the accurate detection of fungus-contaminated agricultural samples.

## 2. Results and Discussion

### 2.1. Antigen Preparation and Immunization

To generate antibodies with high affinity for *F. verticillioides* from maize ear, a representative strain, wh1-2, from a *F. verticillioides* population collected from maize ear rot samples in China was selected for antigen preparation. Species identity and potential fumonisin production were molecularly identified (data not shown). The cell wall-bound proteins (CWPs) were prepared from this fumonisin-producing strain and used to immunize chickens. Indirect ELISA against CWPs with polyclonal antisera from immunized chickens showed a clear robust immune response up to 1:128000, and then mRNA from the spleen cells of chickens was isolated and transcribed into cDNA for construction of phage display library.

### 2.2. Selection of Phage Clones Reactive to Antigens by Phage Display

A phagemid library with a size of 1.4 × 10^7^ clones and 95% clones containing expected inserts was constructed and a good diversity was revealed by *Bst*N I digestion (data not shown). Solid phase panning was employed for screening potential highly reactive scFv antibodies specific to *F. verticillioides*. To select high-affinity scFvs, the number of washing with 0.1% (v/v) PBST and PBS was increased by five (*i.e.*, the first round, 5 times; the second round, 10 times; and the third round, 15 times), while the concentration of coated antigens (CWPs) was decreased by 50 μg/mL from initial concentration of 200 μg/mL in each round of the succeeding panning. Under such conditions, the ratios of output and input phages increased steadily after each round of panning (Table 1), with an approximately 38-fold increase of phage recovery after the third round compared with that from the first round of selection, demonstrating an efficient enrichment of specific antibodies. Thus, the panned library was used for subsequent selection of high affinity antibodies.

### 2.3. Identification of Phage Clones by Phage ELISA

**Figure 1:**
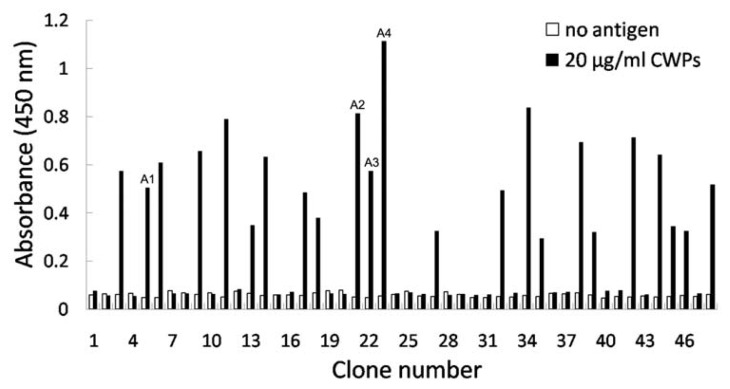
Phage-ELISA based affinity analyses of individual phage clones. Analysis of randomly selected 48 clones from the third round of panning revealed that 23 clones gave a positive reaction to CWPs of *Fusarium verticillioides* with varied signal intensities.

**Figure 2:**
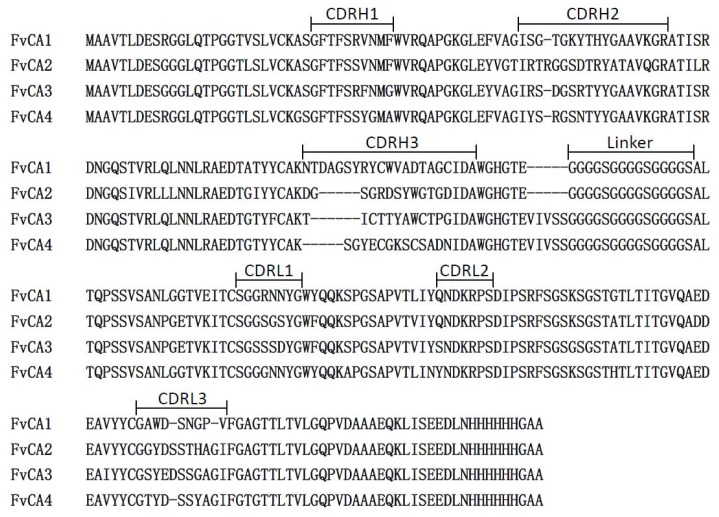
Multiple amino acid sequence alignments of four scFv antibodies specific to *Fusarium verticillioides* selected from Figure 1. The CDRs of the variable domains are indicated. V_H_ and V_L_ fragments are linked with (G_4_S)_3_ linker.

To obtain high affinity scFv antibodies, 48 clones from the panned library were randomly selected and analyzed by phage ELISA. As shown in Figure 1, 23 clones had a positive reaction against CWPs of *F. verticillioides* with varied signal intensities. DNA sequencing of the 10 selected positive clones identified four different scFvs named as FvCA1, FvCA2, FvCA3 and FvCA4, respectively. Sequence analyses indicated that these scFv antibodies contained conserved sequences in their framework regions but had variable sequences in their CDRs, particularly in CDRH3 (Figure 2). They shared 73% sequence identity with each other at their amino acid levels. Their overall conserved sequences and domains were in accordance with chicken antibody sequences in the database of NCBI (data not shown).

### 2.4. Antigen Binding of Soluble Antibodies

**Figure 3:**
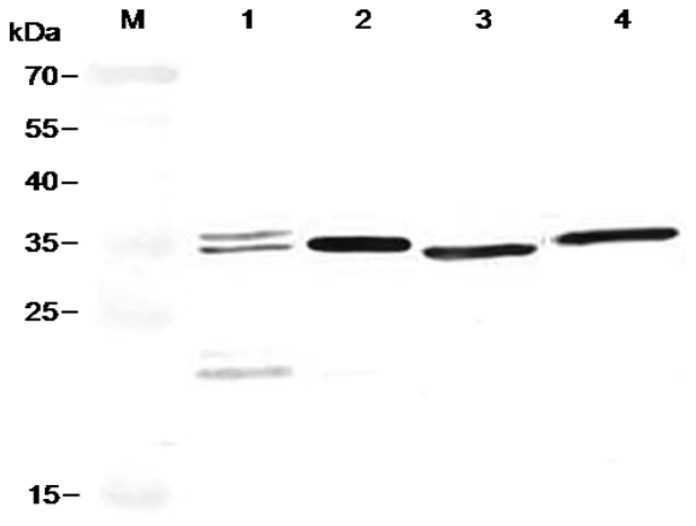
Immunoblot of soluble scFv antibodies extracted from periplasm in *Escherichia coli*. The four selected scFv antibodies were induced for expression by IPTG in *E. coli*. Periplasmic proteins were separated by 12% SDS-PAGE and blotted onto a nitrocellulose membrane. After blocking with 5% (w/v) skimmed milk, the membrane was incubated with a monoclonal anti-His antibody and detected with AP-conjugated goat anti-mouse IgG antibody. The colorimetric reaction was carried out by adding BCIP/NBT substrate. M, protein molecular weight standards. Lanes 1 to 4, FvCA1, FvCA2, FvCA3 and FvCA4 antibodies.

**Figure 4:**
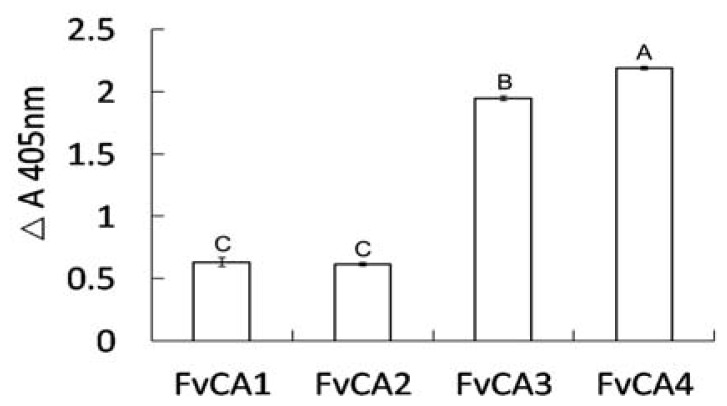
Expression ELISA against CWPs of *Fusarium verticillioides* with bacterially expressed soluble scFv antibodies. Four selected scFv antibodies were analyzed in the presence and absence (control) of the antigens, and the *y* axis represents the OD_405nm_ values minus control values. Values represent mean ± SD of triplicate assays. Letters A, B and C above the column diagram represent a significant difference at *p* < 0.01.

The four selected scFvs expressed in soluble form in bacteria displayed a little size variation as revealed by immunoblot (Figure 3). Three antibodies, FvCA2, FvCA3 and FvCA4, had one intact band while three or four bands were seen for FvCA1 antibody, probably due to incomplete expression or protein degradation. ELISA with four soluble purified scFv antibodies revealed that the FvCA4 had the highest affinity for CWPs of *F. verticillioides*, followed by FvCA3. These two antibodies had a three to four-fold higher binding capacity than the remaining two antibodies, FvCA1 and FvCA2 (Figure 4). The FvCA4 antibody was the best of the four antibodies in both soluble antibody ELISA and phage ELISA. This suggested that phage displayed antibody, especially antibodies with high binding capacity, can be efficiently panned and selected for a given target. However, there were some discrepancies between two ELISAs showing inverse values for FvCA2 and FvCA3, which were antibodies with moderate binding capacity (Figures 1 and 4). FvCA2 had a higher binding value in phage ELISA than FvCA3 but a lower binding was seen in soluble antibody ELISA than for FvCA3. This discrepancy may be due to the alteration of antibody configuration when fused with pIII protein of phage particle, as observed by others [40].

### 2.5. Three-Dimension Structures of scFv Antibodies

**Figure 5:**
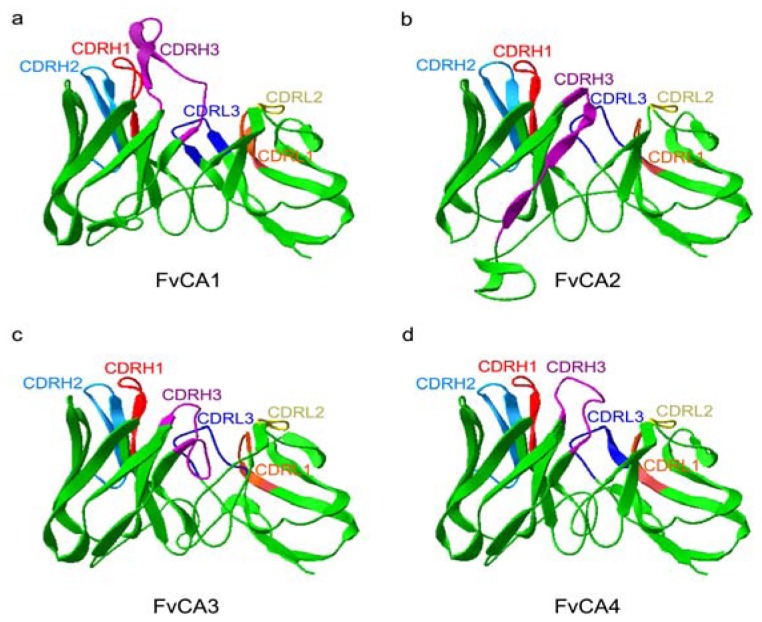
Three-dimension structure modeling of four selected scFv antibodies performed using DeepView. The scFv frameworks and linkers are indicated with green color. The V_H_ and V_L_ domains are shown with their names attached to different colors.

To know whether the three-dimension structures of the selected antibodies have any potential impact on their binding capacity, the amino acid sequences of the four scFvs were subjected to modeling using DeepView (Swiss-PdbViewer) based on the respective crystal structure from CPHmodels-3.2 Server [50] (Figure 5). This modeling analysis indeed revealed a major structural difference among these scFvs in the central cores of protein scaffolds, consisting of CDRH3, CDRL1 and CDRL3 regions. Antibodies FvCA3 and FvCA4 formed a compact structure in this region (Figure 5c,d), while a rather loose configuration was assembled in the structures of FvCA1 and FvCA2 antibodies (Figure 5a,b). Taking into account the different affinities (Figure 4), we supposed that this region might be the centre of the binding site and directly in physical contact with antigen(s), since CDRH3 and CDRL3 were considered to lie generally in the centre of the traditional antigen binding site [51]. Therefore, we could speculate that a compact spatial configuration of paratope is more favorable for the binding of an antibody to CWPs from *F. verticillioides*. The other regions, such as CDRH1, CDRH2 and CDRL2, seemed to have less impact on the antigen-antibody interactions because they reside in the periphery of protein scaffolds.

Notably, most variation in structures was mainly caused by CDRH3 region (Figure 5), in which there was the most diversity of amino acid and length among the six CDRs (Figure 2). For example, FvCA1 had the longest amino acid, and FvCA2 formed a β-turn; FvCA4 exposed its CDRH3 region more closely to the surface compared to that from FvCA3. Therefore, we hypothesized that the residues of CDRH3 played a vital role in these selected antibodies. Previous studies have also confirmed that the length and heterogeneity of the CDRH3 sequence were associated to antibody binding affinity, specificity, paratope shape, and activity (such as neutralization potency) [52–56]. Properties of the selected scFvs such as FvCA1, FvCA2 or FvCA3 might be further improved by CDR shuffling [57–59], chain shuffling [60], or DNA shuffling technology [39,40].

### 2.6. Characterization of FvCA4 Antibody

**Figure 6:**
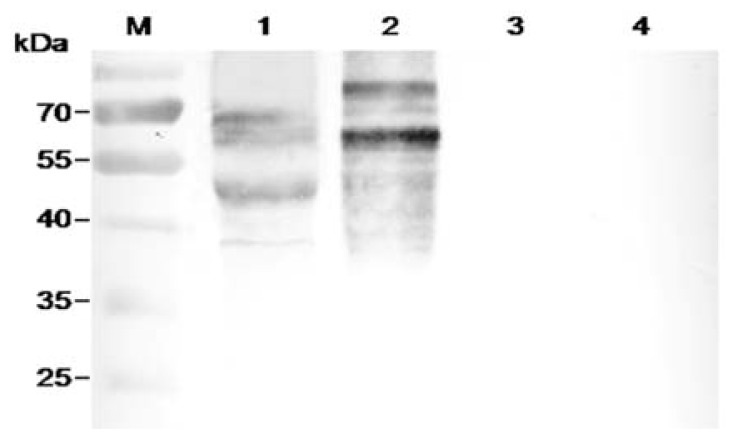
Immunoblot of CWPs from different fungal species detected with FvCA4 antibody. Twenty microliters of CWPs (about 50 to 100 ng of total protein) were loaded on a 12% SDS-PAGE gel, separated and blotted onto nitrocellulose membrane. After blocking with 5% (w/v) skimmed milk, the membrane was incubated with 50 nmol/L bacterially expressed FvCA4 followed by the addition of a mouse anti-His antibody and a AP-conjugated goat anti-mouse IgG antibody. The colorimetric reaction was carried out by adding BCIP/NBT substrate. M, protein molecular weight standards. Lanes 1 to 4, the CWPs of *Fusarium verticillioides*, *Fusarium asiaticum*, *Aspergillus flavus*, and *Sclerotinia sclerotiorum*.

The above analyses indicated that the FvCA4 antibody had the best binding capacity and proper overall three-dimensional structure. Thus, this antibody was selected for further characterization in its soluble form. The germinated conidiospores of different *Fusarium* species and other fungi were tested for this antibody by ELISA. As shown in Table 2, FvCA4 antibody had a high sensitivity to all *Fusarium* species but no cross-reactivity with non-*Fusarium* fungi, suggesting that FvCA4 is a *Fusarium*-specific antibody. To further reveal whether FvCA4 antibody was able to react with the components from CWPs of this fungus, an immunoblot assay was carried out with the CWPs from *F. verticillioides*, *F. asiaticum*, *Aspergillus flavus*, and *Sclerotinia sclerotiorum*. The results indicated that this antibody indeed bound to components from CWPs of *Fusarium* species but did not cross-react with that of *A. flavus* and *S. sclerotiorum* (Figure 6). Different sizes of CWPs from *F. verticillioides* and *F. asiaticum* detected in the blot suggested that these two *Fusarium* species have varied cell wall compositions as they belong to different species and have their own favorable hosts and peculiar infection mechanisms. Nevertheless, these results congruously indicated that FvCA4 is a *Fusarium* genus-specific antibody.

### 2.7. Localization Labeling of FvCA4 Antibody Binding

**Figure 7:**
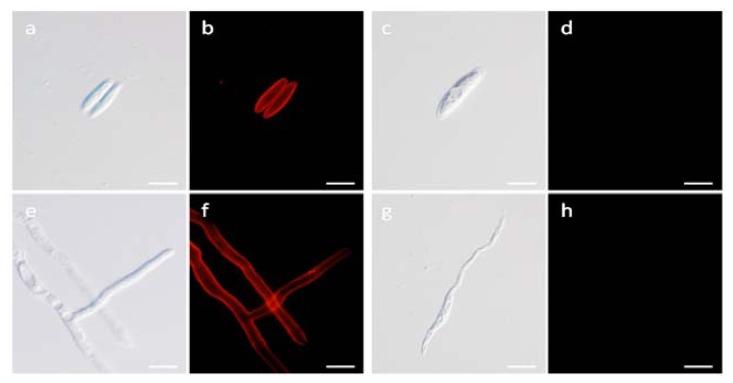
Photomicrographs of *Fusarium verticillioides* immunostained with FvCA4 antibody. (**a**) and (**c**), conidiospores of *F. verticillioides* examined under a bright-field microscope; (**e**) and (**g**), hyphae of *F. verticillioides* examined under a bright-field microscope; (**b**) and (**f**), the same slides shown in panel (**a**) and (**e**) were examined under Cy3 with FvCA4 antibody; (**d**) and (**h**), the same slides shown in panel (**c**) and (**g**) were examined under Cy3 with PIPP antibody. Bar, 10 μm.

Immunofluorescence labeling was used to identify the site of FvCA4 binding. Figure 7a,c,e,g show the normal morphology of conidiospores and hyphae of *F. verticillioides*. Figure 7b,f show the binding of FvCA4 localized with the brightest fluorescence intensity to the cell walls of both conidiospores and hyphae, confirming the specificity of this antibody for a cell surface target. No fluorescence labeling was visible after incubation with the nonspecific scFv antibody PIPP specific to a human antigen HCG [32,40,61] (Figure 7d,h). These results confirmed that FvCA4 binds to a cell surface antigen of *F. verticillioides*.

### 2.8. Immunological Detection of Mycelium and Contaminated Samples

**Figure 8:**
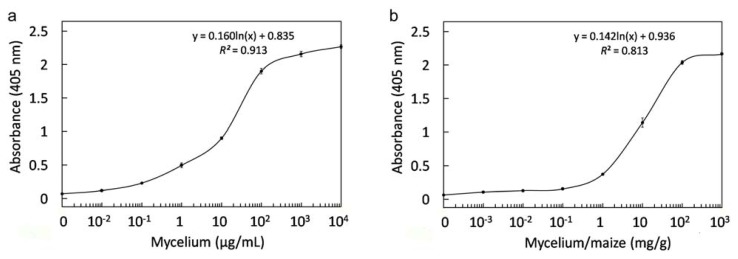
(**a**) The detection limit to mycelium of FvCA4 antibody was determined with different concentrations of *Fusarium verticillioides* mycelium; (**b**) The minimum detectable quantity of mycelium in maize grains was determined. An aliquot of 100 μL mycelium or maize-mycelium mixture at indicated concentrations was added into plate wells for ELISAs detection with 200 nmol/L purified scFv antibody. Values represent mean ± SD of triplicate assays.

To study the feasibility of FvCA4 for detection of contaminated samples, various concentrations of mycelium were used for ELISAs to determine detection limit. Twice higher optical density of samples to controls was considered as valid detection [62] and the result showed that the FvCA4 antibody was able to detect as low as approximately 10^−2^ μg/mL of *F. verticillioides* mycelium (Figure 8a). The mycelium concentration in relation to the OD_405nm_ values could be expressed by logarithmic curve [y = 0.160ln(x) + 0.835, *R^2^* = 0.913]. To determine the minimum level of mycelium biomass present in maize grains, mycelium was mixed with maize grains and the extracts were used for ELISAs. Figure 8b shows that FvCA4 was able to detect approximately 10^−2^ mg mycelium per gram of maize grains (*i.e.*, 0.1 μg/mL). The detection limit of mycelium was higher in maize grains than in PBS buffer, as observed by others [24]. Logarithmic curve regularity between mycelium biomass per gram of maize and the OD_405nm_ values was illustrated as follows: [y = 0.142ln(x) + 0.936, *R^2^* = 0.813]. Therefore, the mycelium biomass in contaminated food/feed can be assessed conveniently by an ELISA detection using the FvCA4 antibody.

To study the stability of antigens detected by the antibody, naturally contaminated and artificially infected maize samples were heated to 100 °C for 10 min and assayed by an ELISA. The results indicated that comparable reaction signals were detected from both heated and unheated samples. These results suggested that the antigens present in *F. verticillioides* are thermostable and FvCA4 can efficiently detect contaminated field samples and processed food products.

## 3. Experimental Section

### 3.1. Antigen Preparation and Immunization

A fungus strain of *F. verticillioides*, wh1-2, was isolated from the rot kernels of maize, collected from the seriously epidemic area of maize ear rot in Wuhan, China. This strain was identified by PCR with genus- and fumonisin-producing-specific primers [63] and cultured in Czapek-Dox Broth medium (pH 8.0) at 30 °C with shaking at 200 r/min for 5 days. The mycelia were collected and freeze-dried, and the cell wall-bound proteins (CWPs) were prepared as described [64].

For immunization, 12 week-old “White leghorn” chickens (*Gallus domesticus*) were injected intramuscularly with 100 μg antigen emulsified with an equal-volume complete Freund’s adjuvant (Sigma). Three additional injections were given at 2-week intervals with an equal-volume of incomplete Freund’s adjuvant (Sigma). The blood was collected after the third immunization and the antisera titer was measured by indirect ELISA [25]. Briefly, 96-well microtiter plate wells were coated with 100 μL CWPs (20 μg/mL) and blocked with 150 μL 2% (w/v) skimmed milk in PBS. Wells were washed three times with PBS, followed by incubation with 2-fold diluted immune and non-immune sera from 1:1000 initial dilution for 1.5 h at 37 °C. Then ELISA detection was performed with 1:5000 diluted alkaline phosphatase (AP)-conjugated anti-chicken IgY (Promega) and the enzyme reaction was activated by 0.2% (w/v) *p*-nitrophenyl phosphate (pNPP) substrate solution. The absorbance at 405 nm was measured on an ELISA Microplate Reader (Multiskan MK3, Thermo Fisher).

### 3.2. Library Construction

Total RNA was isolated from the spleen cells collected after the fourth immunization using TRNzol-A+ total RNA extraction reagent (Tiangen) and mRNA was purified using Oligotex mRNA mini kit (Qiagen) following the Manufacturer’s instructions. The first strand of V_H_ and V_L_ cDNAs were synthesized with VH-cDNA and VL-cDNA primers (Table 3) respectively, using the SuperScript III Reverse Transcriptase Kit (Invitrogen). Subsequently, V_H_ domains were amplified by PCR using sense primer VHF and antisense primer CHIC-Gly, as well as the CHIC-Ser and VLB primers (Table 3) for V_L_ domains amplification. The scFv fragments were then assembled with purified V_H_ and V_L_ domains by SOE-PCR (splicing by overhang extension-PCR) using VHF and VLB primers.

After SOE-PCR reaction, the full-size scFv genes were purified and digested with *Sfi*I and *Not* I restriction enzymes (NEB), and cloned into the pHENHi phagemid vector [32] with the same restriction enzyme sites. The resulting recombinant phagemids were then transformed into *E. coli* XL1-Blue MRF’ (Stratagene) by electroporation. The transformed cells were plated onto Luria-Bertani agar medium supplemented with 1% (w/v) glucose and 100 μg/mL ampicillin. All clones were scraped from the plates in Luria-Bertani broth containing 100 μg/mL ampicillin and 25% (v/v) glycerol and stored at −70 °C.

### 3.3. Panning

An aliquot of 800 μL recombinant *E. coli* cells of the constructed library was inoculated into 50 mL of fresh 2 × TY broth (1.6% (w/v) tryptone, 1.0% (w/v) yeast extract, 0.5% (w/v) NaCl) supplemented with 1% (w/v) glucose and 100 μg/mL ampicillin and incubated at 37 °C with shaking at 200 r/min until the OD_600nm_ reached 0.5. Approximately 5 × 10^10^ M13-KO7 helper phages (Amersham Biosciences) were added into 5 mL culture and incubated at 37 °C for 30 min without shaking. After centrifugation, the infected cells were resuspended in 140 mL 2 × TY broth containing 100 μg/mL ampicillin and 25 μg/mL kanamycin and cultured at 30 °C for 16 h with shaking at 200 r/min. The recombinant phages were recovered by precipitation with PEG/NaCl as described [65].

Solid phase panning was carried out according to Andris-Widhopf *et al*. [42] with modifications. Briefly, 20 wells of microtiter plate were coated with CWPs (200 μg/mL in sterile PBS and 50 μg/mL descending in the succeeding rounds of panning) at 37 °C for 2 h. After blocking with 2% (w/v) skimmed milk in PBS, 100 μL of the freshly prepared phages suspension was added to each well and incubated at 37 °C for at least 2 h. The wells were washed five times (10 and 15 washing times in the second and third round of panning) with PBST (0.1% (v/v) Tween-20 in PBS) and PBS. The bound phages were eluted by applying 100 μL 100 mM triethylamine at room temperature for 10 min without shaking. Then, 50 μL Tris-HCl (1 mol/L, pH 7.4) was added immediately for quick neutralization. The log-phase bacteria infected with eluted phages were plated onto TYE-GA agar plates (1.0% (w/v) tryptone, 0.5% (w/v) yeast extract, 0.8% (w/v) NaCl, 1% (w/v) glucose, and 100 μg/mL ampicillin) and incubated at 37 °C overnight. The clones were collected for the next round of panning. In the panning procedure, one well was blocked with 2% (w/v) skimmed milk and 10 μL of prepared phages was added to serve as a control.

### 3.4. Phage ELISA

The 96-well microtiter plates were coated with 100 μL CWPs (20 μg/mL) or PBS (control) and blocked with 150 μL 2% (w/v) skimmed milk at 37 °C for 2 h. 50 μL of the blocking solution and 50 μL of phages prepared from the clones of the third round of panning were added into wells and incubated for 2 h at 37 °C. After three washings with 0.1% (v/v) PBST and PBS, the bound phages were detected with 1:5000 diluted HRP-conjugated mouse anti-M13 antibody (GE Healthcare) at 37 °C. The 3,3’,5,5’-tetramethylbenzidine (TMB) substrate solution was added to each well and the plates were incubated for 15 min for color development. The reaction was stopped with 2 mol/L H_2_SO_4_ and absorbance at 450 nm was measured.

### 3.5. Sequencing

The positive clones identified in phage ELISA were selected for DNA sequencing (Invitrogen). The forward primer pHENpel and the reverse primer pHENmyc (Table 3) were designed based on the pHENHi phagemid vector and used for sequencing scFv genes. Sequences were analyzed using BioEdit software (Ibis Biosciences).

### 3.6. Expression and Purification

The colony culture from a −70 °C glycerol stock was inoculated into 20 mL 2 × TY broth containing 1% (w/v) glucose and 100 μg/mL ampicillin and cultured at 37 °C overnight with shaking at 200 r/min. The next day, an aliquot of 8 mL of the bacteria culture was transferred into 160 mL 2 × TY broth with ampicillin and grown to an OD_600nm_ of 0.5 to 0.6. A final concentration of 0.1 mmol/L IPTG was then added into the culture and the cells were incubated at 25 °C and 200 r/min for 2 h for scFv expression. The periplasmic proteins were extracted by osmotic shock method [40,66] and dialyzed against PBS buffer. The soluble scFv antibodies were purified by immobilized metal affinity chromatography (IMAC, Qiagen).

### 3.7. ELISA Assays

For affinity analysis of scFv antibodies, 96-well microtiter plates were coated with 100 μL 20 μg/mL CWPs in PBS and blocked with 150 μL 2% (w/v) skimmed milk at 37 °C for 2 h. After three washings with PBS, 200 nmol/L of purified scFv antibody was added and incubated for 1.5 h at 37 °C. Subsequently, the plate wells were incubated with 100 μL 1:5000 diluted monoclonal anti-polyhistidine (His) antibody (Sigma) and AP-conjugated goat anti-mouse IgG antibody (Sigma) for 1.5 h at 37 °C. Colorimetric detection was performed with 0.2% (w/v) pNPP substrate solution and absorbance at 405 nm was measured. For analysis of specificity, the wells were coated with germinated conidiospores (10^7^/mL) of *Fusarium* and other genera fungi in YPG broth (0.3% (w/v) yeast extract, 1% (w/v) peptone, and 2% (w/v) glucose), and then ELISA detection was performed as described above. The YPG broth and PBS were taken as control separately and each sample was done in triplicate.

### 3.8. Immunoblot Analyses

The CWPs from *F. verticillioides*, *F. asiaticum*, *Aspergillus flavus*, and *Sclerotinia sclerotiorum* or the periplasmic proteins from scFv expression system were separated by 12% (w/v) SDS-PAGE and transferred to nitrocellulose membranes. Immunoblot containing CWPs was developed using the purified scFv antibody (50 nmol/L), a 1:5000 diluted monoclonal anti-His antibody and AP-conjugated goat anti-mouse IgG antibody. The membrane with periplasmic proteins was detected with the antibodies described above, omitting the scFv antibody. The colorimetric reaction was carried out by using the BCIP/NBT Color Development Kit (Boster). The immunoblot membranes were scanned with a Bio-Rad GS-800 Densitometer.

### 3.9. Immunofluorescence Microscopy

The wells of a 12-well cell culture plate (Corning) were blocked with 2% (w/v) BSA in PBS at 37 °C for 2 h followed by three washings with PBS. The poly-l-lysin covered nummular cover slides were placed into the wells and 1 mL of germinated conidiospores was added. The supernatant was discarded after centrifugation at 2000 g for 15 min. 500 μL of the purified scFv (1 μg/mL), monoclonal anti-His antibody (1:3000 dilution in PBS) and Cy3-conjugated affinipure goat anti-mouse IgG (H + L) (1:100 dilution, ProteinTech Group) was added in turn. Unbound antibodies were washed away with 0.1% (v/v) PBST and PBS each three times. Then the prepared coverslips were transferred with the embedded antigen down on the slides by adding Antifade Mounting Medium (Beyotime). A Nikon Eclipse 90i fluorescence microscope with a TRITC model and the NIS-Elements AR 3.2 software (version 3.2; Nikon: Tokyo, Japan, 2011) was used for microscopic detection and analysis.

### 3.10. Sample Preparation for Immunological Detection

To obtain the detection limit to mycelium, different concentrations of *F. verticillioides* mycelium ground in PBS (10^−2^, 10^−1^, 1, 10, 10^2^, 10^3^, 10^4^ μg/mL) were added into plate wells and ELISA detection was performed as described above. For determining the minimum level of mycelium biomass present in maize grains, 0.1 g of maize powder suspended in 10 mL PBS containing 0.05% (v/v) Tween-20 was mixed with different amounts of mycelium (10^−3^, 10^−2^, 10^−1^, 1, 10, 10^2^, 10^3^ mg). Then, 100 μL aliquot of the homogenates was added into plate wells for ELISA detection.

To determine the feasibility of *Fusarium* contamination detection using the bacteria expressed scFv antibody, 10 naturally and artificially infected maize samples were collected from different areas of China and ground in liquid nitrogen. The powder was mixed vigorously in PBS and the supernatants were added into plate wells. The test assay was developed as described for ELISA assays. For thermostability analysis, the molded samples were treated by roasting or boiling at 100 °C for 10 min. Also, healthy samples were similarly treated to serve as negative controls and each sample was done in triplicate.

## 4. Conclusions

In this study, we isolated, for the first time, highly reactive scFv antibodies against *F. verticillioides* from immunized chickens by phage display. Both phage ELISA and soluble antibody ELISA identified that one scFv antibody, FvCA4, displayed the highest binding capacity. Structural modeling revealed a favorable three-dimensional structure of paratope for the FvCA4 antibody, with CDRH3, CDRL1 and CDRL3 being centered in the core region of the antibody surface. Other scFv antibodies may be altered by molecular evolution, especially in their CDRH3, to improve their binding properties. Specificity and reactivity assays indicated that the FvCA4 antibody had a binding specificity to all *Fusarium* species and had no cross-reactivity with non-*Fusarium* species. Immunoblot analysis showed that the FvCA4 reacted to different components from CWPs of *F. verticillioides* and *F. asiaticum*, respectively. Immunofluoresence labeling confirmed the binding of FvCA4 localized to the cell walls of conidiospores and hyphae of *F. verticillioides*, demonstrating the specificity of this antibody for a cell surface target. Moreover, the FvCA4 antibody was able to detect mycelium at a concentration as low as 10^−2^ μg/mL and mycelium biomass contamination in maize at a quantity of 10^−2^ mg/g (*i.e.*, 0.1 μg/mL). This antibody also bound to naturally contaminated samples that were roasted or boiled. Taken together, the isolated scFv antibody shows a promising application for rapid and accurate detection of *Fusarium* species as well as *Fusarium* contaminated agricultural samples and processed food products.

## Figures and Tables

**Table 1:** Phages applied and eluted in each round of panning by phage display.

Round	Input (pfu)	Output (pfu)	Ratio (%)
1	2.6 × 10^12^	1.5 × 10^4^	5.8 × 10^−9^
2	4.5 × 10^12^	6.0 × 10^4^	1.3 × 10^−8^
3	5.4 × 10^12^	1.2 × 10^6^	2.2 × 10^−7^

pfu, plaque-forming unit.

**Table 2:** Reactivity and specificity of FvCA4 antibody determined by ELISA detection.

Species	Host	Reactivity of FvCA4
*F. verticillioides*	maize	+++
*F. verticillioides*	rice	+++
*F. proliferatum*	maize	+++
*F. proliferatum*	potato	+++
*F. proliferatum*	wheat	+++
*F. oxysporum* f.sp. *matthiolae*	stock	+++
*F. oxysporum* f.sp. *zingiberi*	turmeric	+++
*F. oxysporum* f.sp. *niveum*	watermelon	+++
*F. poae*	wheat	+++
*F. culmorum*	wheat	+++
*F. asiaticum*	wheat	++
*F. solani*	mung bean	+++
*F. tricinctum*	wheat	+++
*A. flavus*	peanut	−
*S. sclerotiorum*	rapeseed	−
*R. cerealis*	wheat	−
*M. fructicola*	peach	−
*V. dahliae*	cotton	−

The wells were coated with fresh mycelium of different fungal species as described above. Colorimetric reactions were done upon adding *p*-nitrophenyl phosphate substrate and measured at 405 nm. The scale presents an arbitrary set of OD_405nm_ readings (<0.1 OD, −; 0.1–0.8 OD, +; 0.801–1.6 OD, ++; >1.601 OD, +++) after 30 min substrate reaction.

**Table 3:** Nucleotide sequences of primers used in PCR amplification and DNA sequencing.

Name	Nucleotide Sequence
VH-cDNA	5’-CGGTGGGGGACATCTGAGTGGG-3’
VL-cDNA	5’-AGGGGTGGAGGACCTGCACCTC-3’
CPDVHF	5’-ATCTAGGCATCCCTTGGCCCAGCCGGCCATGGCTGCCGTGACGTTGGACGAGTCC-3’
CHIC-Gly	5’-GCCAGAGCCACCTCCGCCTGAACCGCCTCCACCTGAGGAGACGATGACCTCGGTCCC-3’
CHIC-Ser	5’-GGCGGAGGTGGCTCTGGCGGTGGCGGATCGGCGCTGACTCAGCCGTCCTCGGTG-3’
CPDVLB	5’-TGACCTGCGAGGATGCGCGGCCGCGTCGACGGGCTGGCCTAGGACGGTCAG-3’
pHENpel	5’-GCAGCCGCTGGATTGTTATTACTCGC-3’
pHENmyc	5’-ATTCAGATCCTCTTCTGAGATGAG-3’

## References

[1] Bacon C.W., Williamson J.W. (1992). Interactions of *Fusarium moniliforme*, its metabolites and bacteria with corn. Mycopathologia.

[2] Desjardins A.E. (2003). *Gibberella* from A (venaceae) to Z (eae). Annu. Rev. Phytopathol.

[3] Nelson P.E., Desjardins A.E., Plattner R.D. (1993). Fumonisins, mycotoxins produced by *Fusarium* species: Biology, chemistry, and significance. Annu. Rev. Phytopathol.

[4] Wang E., Norred W.P., Bacon C.W., Riley R.T., Merrill A.H. (1991). Inhibition of sphingolipid biosynthesis by fumonisins. Implications for diseases associated with *Fusarium moniliforme*. J. Biol. Chem..

[5] Yoo H.S., Norred W.P., Wang E., Merrill A.H., Riley R.T. (1992). Fumonisin inhibition of *de novo* sphingolipid biosynthesis and cytotoxicity are correlated in LLC-PK1 cells. Toxicol. Appl. Pharmacol..

[6] Voss K.A., Plattner R.D., Riley R.T., Meredith F.I., Norred W.P. (1998). *In vivo* effects of fumonisin B_1_-producing and fumonisin B_1_-nonproducing *Fusarium moniliforme* isolates are similar: Fumonisins B_2_ and B_3_ cause hepato- and nephrotoxicity in rats. Mycopathologia.

[7] Voss K.A., Riley R.T., Norred W.P., Bacon C.W., Meredith F.I., Howard P.C., Plattner R.D., Collins T.F., Hansen D.K., Porter J.K. (2001). An overview of rodent toxicities: Liver and kidney effects of fumonisins and *Fusarium moniliforme*. Environ. Health Perspect.

[8] Odhav B., Adam J.K., Bhoola K.D. (2008). Modulating effects of fumonisin B1 and ochratoxin A on leukocytes and messenger cytokines of the human immune system. Int. Immunopharmacol.

[9] Stockmann-Juvala H., Alenius H., Savolainen K. (2008). Effects of fumonisin B_1_ on the expression of cytokines and chemokines in human dendritic cells. Food Chem. Toxicol.

[10] Carlson D.B., Williams D.E., Spitsbergen J.M., Ross P.F., Bacon C.W., Meredith F.I., Riley R.T. (2001). Fumonisin B_1_ promotes aflatoxin B_1_ and *N*-methyl-*N′*-nitro-nitrosoguanidine-initiated liver tumors in rainbow trout. Toxicol. Appl. Pharmacol.

[11] Sun G., Wang S., Hu X., Su J., Zhang Y., Xie Y., Zhang H., Tang L., Wang J.S. (2011). Co-contamination of aflatoxin B_1_ and fumonisin B_1_ in food and human dietary exposure in three areas of China. Food Addit. Contam. A.

[12] Rheeder J.P., Marasas W.F.O., Thiel P.G., Sydenham E.W., Shephard G.S., van Schalkwyk D.J. (1992). *Fusarium moniliforme* and fumonisins in corn in relation to human esophageal cancer in Transkei. Phytopathology.

[13] Myburg R.B., Dutton M.F., Chuturgoon A.A. (2002). Cytotoxicity of fumonisin B_1_, diethylnitrosamine, and catechol on the SNO esophageal cancer cell line. Environ. Health Perspect.

[14] Sun G., Wang S., Hu X., Su J., Huang T., Yu J., Tang L., Gao W., Wang J.S. (2007). Fumonisin B_1_ contamination of home-grown corn in high-risk areas for esophageal and liver cancer in China. Food Addit. Contam.

[15] Ueno Y., Iijima K., Wang S.D., Sugiura Y., Sekijima M., Tanaka T., Chen C., Yu S.Z. (1997). Fumonisins as a possible contributory risk factor for primary liver cancer: A 3-year study of corn harvested in Haimen, China, by HPLC and ELISA. Food Chem. Toxicol.

[16] Yang H.Q., Lu F.Y., Hao Y.K., Dong B. (2011). Situation analysis and development strategy of maize industry in China. Chin. Agric. Sci. Bull.

[17] Wang Z., Liu X., Cong L., Li X., Tong Z., Cheng S., Ge S. (2000). Characteristics of producing-fumonisin and dimorphic fungus of *Fusarium moniliforme*. Chin. J. Prev. Med.

[18] Gong H.Z., Ji R., Li Y.X., Zhang H.Y., Li B., Zhao Y., Sun L., Yu F., Yang J. (2009). Occurrence of fumonisin B_1_ in corn from the main corn-producing areas of china. Mycopathologia.

[19] Parsons M.W., Munkvold G.P. (2010). Associations of planting date, drought stress, and insects with *Fusarium* ear rot and fumonisin B_1_ contamination in California maize. Food Addit. Contam. A.

[20] Samapundo S., Devliehgere F., de Meulenaer B., Debevere J. (2005). Effect of water activity and temperature on growth and the relationship between fumonisin production and the radial growth of *Fusarium verticillioides* and *Fusarium proliferatum* on corn. J. Food Prot.

[21] Miller J.D. (2001). Factors that affect the occurrence of fumonisin. Environ. Health Perspect.

[22] Notermans S., Heuvelman C.J. (1985). Immunological detection of moulds in food by using the enzyme-linked immunosorbent assay (ELISA); preparation of antigens. Int. J. Food Microbiol.

[23] Lin H.H., Cousin M.A. (1987). Evaluation of enzyme-linked immunosorbent assay for detection of molds in foods. J. Food Sci.

[24] Iyer M.S., Cousin M.A. (2003). Immunological detection of *Fusarium* species in cornmeal. J. Food Prot.

[25] Biazon L., Meirelles P.G., Ono M.A., Itano E.N., Taniwaki M.H., Sugiura Y., Ueno Y., Hirooka E.Y., Ono E.Y.S. (2006). Development of polyclonal antibodies against *Fusarium verticillioides* exoantigens. Food Agric. Immunol.

[26] De Ruiter G.A., van Bruggen-van der Lugt A.W., Bos W., Notermans S.H., Rombouts F.M., Hofstra H. (1993). The production and partial characterization of a monoclonal IgG antibody specific for moulds belonging to the order Mucorales. J. Gen. Microbiol.

[27] Hiatt E.E., Hill N.S., Hiatt E.N. (2001). Monoclonal antibodies incorporated into *Neotyphodium coenophialum* fungal cultures: Inhibition of fungal growth and stability of antibodies. Fungal Genet. Biol..

[28] Kwak B.Y., Shon D.H., Kwon B.J., Kweon C.H., Lee K.H. (2001). Detection of *Aspergillus* and *Penicillium* genera by enzyme-linked immunosorbent assay using a monoclonal antibody. J. Microbiol. Biotechnol.

[29] Thornton C.R., Slaughter D.C., Davis R.M. (2010). Detection of the sour-rot pathogen *Geotrichum candidum* in tomato fruit and juice by using a highly specific monoclonal antibody-based ELISA. Int. J. Food Microbiol.

[30] Yajima W., Rahman M.H., Das D., Suresh M.R., Kav N.N. (2008). Detection of *Sclerotinia sclerotiorum* using a monomeric and dimeric single-chain fragment variable (scFv) antibody. J. Agric. Food Chem.

[31] Yang B., Yajima W., Das D., Suresh M.R., Kav N.N. (2009). Isolation, expression and characterization of two single-chain variable fragment antibodies against an endo-polygalacturonase secreted by *Sclerotinia sclerotiorum*. Protein Expr. Purif.

[32] Peschen D., Li H.P., Fischer R., Kreuzaler F., Liao Y.C. (2004). Fusion proteins comprising a *Fusarium*-specific antibody linked to antifungal peptides protect plants against a fungal pathogen. Nat. Biotechnol.

[33] Li H.P., Zhang J.B., Shi R.P., Huang T., Fischer R., Liao Y.C. (2008). Engineering Fusarium head blight resistance in wheat by expression of a fusion protein containing a *Fusarium*-specific antibody and an antifungal peptide. Mol. Plant Microbe Interact.

[34] Yajima W., Verma S.S., Shah S., Rahman M.H., Liang Y., Kav N.N. (2010). Expression of anti-sclerotinia scFv in transgenic *Brassica napus* enhances tolerance against stem rot. N. Biotechnol.

[35] Hu Z.Q., Li H.P., Zhang J.B., Glinka E., Liao Y.C. (2008). Antibody-mediated prevention of *Fusarium* mycotoxins in the field. Int. J. Mol. Sci.

[36] Safarnejad M.R., Jouzani G.S., Tabatabaie M., Twyman R.M., Schillberg S. (2011). Antibody-mediated resistance against plant pathogens. Biotechnol. Adv.

[37] Winter G., Griffiths A.D., Hawkins R.E., Hoogenboom H.R. (1994). Making antibodies by phage display technology. Annu. Rev. Immunol.

[38] Holliger P., Hudson P.J. (2005). Engineered antibody fragments and the rise of single domains. Nat. Biotechnol.

[39] Fukuda I., Kojoh K., Tabata N., Doi N., Takashima H., Miyamoto-Sato E., Yanagawa H. (2006). *In vitro* evolution of single-chain antibodies using mRNA display. Nucleic Acids Res.

[40] Liu J.L., Hu Z.Q., Xing S., Xue S., Li H.P., Zhang J.B., Liao Y.C. (2011). Attainment of 15-fold higher affinity of a *Fusarium*-specific single-chain antibody by directed molecular evolution coupled to phage display. Mol. Biotechnol.

[41] Davies E.L., Smith J.S., Birkett C.R., Manser J.M., Anderson-Dear D.V., Young J.R. (1995). Selection of specific phage-display antibodies using libraries derived from chicken immunoglobulin genes. J. Immunol. Methods.

[42] Andris-Widhopf J., Rader C., Steinberger P., Fuller R., Barbas C.F. (2000). Methods for the generation of chicken monoclonal antibody fragments by phage display. J. Immunol. Methods.

[43] Yamanaka H.I., Inoue T., Ikeda-Tanaka O. (1996). Chicken monoclonal antibody isolated by a phage display system. J. Immunol.

[44] Foord A.J., Muller J.D., Yu M., Wang L.F., Heine H.G. (2007). Production and application of recombinant antibodies to foot-and-mouth disease virus non-structural protein 3ABC. J. Immunol. Methods.

[45] Reynaud C.A., Anquez V., Grimal H., Weill J.C. (1987). A hyperconversion mechanism generates the chicken light chain preimmune repertoire. Cell.

[46] Reynaud C.A., Dahan A., Anquez V., Weill J.C. (1989). Somatic hyperconversion diversifies the single V_H_ gene of the chicken with a high incidence in the D region. Cell.

[47] McCormack W.T., Tjoelker L.W., Thompson C.B. (1993). Immunoglobulin gene diversification by gene conversion. Prog. Nucleic Acid Res. Mol. Biol.

[48] Woolley J.A., Landon J. (1995). Comparison of antibody production to human interleukin-6 (IL-6) by sheep and chickens. J. Immunol. Methods.

[49] Svendsen Bollen L., Crowley A., Stodulski G., Hau J. (1996). Antibody production in rabbits and chickens immunized with human IgG. A comparison of titre and avidity development in rabbit serum, chicken serum and egg yolk using three different adjuvants. J. Immunol. Methods.

[50] CPHmodels 3.2 Server.

[51] Duquesnoy R.J. (2006). A structurally based approach to determine HLA compatibility at the humoral immune level. Hum. Immunol.

[52] Ohno S., Mori N., Matsunaga T. (1985). Antigen-binding specificities of antibodies are primarily determined by seven residues of V_H_. Proc. Natl. Acad. Sci. USA.

[53] Wu T.T., Johnson G., Kabat E.A. (1993). Length distribution of CDRH3 in antibodies. Proteins.

[54] Barrios Y., Jirholt P., Ohlin M. (2004). Length of the antibody heavy chain complementarity determining region 3 as a specificity-determining factor. J. Mol. Recognit.

[55] Kim M., Sun Z.Y., Rand K.D., Shi X., Song L., Cheng Y., Fahmy A.F., Majumdar S., Ofek G., Yang Y. (2011). Antibody mechanics on a membrane-bound HIV segment essential for GP41-targeted viral neutralization. Nat. Struct. Mol. Biol.

[56] Nuttall S.D., Irving R.A., Hudson P.J. (2000). Immunoglobulin V_H_ domains and beyond: Design and selection of single-domain binding and targeting reagents. Curr. Pharm. Biotechnol.

[57] Jirholt P., Ohlin M., Borrebaeck C.A., Soderlind E. (1998). Exploiting sequence space: Shuffling *in vivo* formed complementarity determining regions into a master framework. Gene.

[58] Jirholt P., Strandberg L., Jansson B., Krambovitis E., Soderlind E., Borrebaeck C.A., Carlsson R., Danielsson L., Ohlin M. (2001). A central core structure in an antibody variable domain determines antigen specificity. Protein Eng.

[59] Schoonbroodt S., Steukers M., Viswanathan M., Frans N., Timmermans M., Wehnert A., Nguyen M., Ladner R.C., Hoet R.M. (2008). Engineering antibody heavy chain CDR3 to create a phage display Fab library rich in antibodies that bind charged carbohydrates. J. Immunol.

[60] Kang A.S., Jones T.M., Burton D.R. (1991). Antibody redesign by chain shuffling from random combinatorial immunoglobulin libraries. Proc. Natl. Acad. Sci. USA.

[61] Kathuria S., Sriraman R., Nath R., Sack M., Pal R., Artsaenko O., Talwar G.P., Fischer R., Finnern R. (2002). Efficacy of plant-produced recombinant antibodies against HCG. Hum. Reprod.

[62] Rodoni B.C., Dale J.L., Harding R.M. (1999). Characterization and expression of the coat protein-coding region of banana bract mosaic potyvirus, development of diagnostic assays and detection of the virus in banana plants from five countries in southeast Asia. Arch. Virol.

[63] Patiño B., Mirete S., González-Jaén M.T., Mulé G., Rodríguez M.T., Vázquez C. (2004). PCR detection assay of fumonisin-producing *Fusarium verticillioides* strains. J. Food. Prot.

[64] Schoffelmeer E.A., Klis F.M., Sietsma J.H., Cornelissen B.J. (1999). The cell wall of *Fusarium oxysporum*. Fungal Genet. Biol.

[65] Kirsch M., Zaman M., Meier D., Dubel S., Hust M. (2005). Parameters affecting the display of antibodies on phage. J. Immunol. Methods.

[66] Nossal N.G., Heppel L.A. (1966). The release of enzymes by osmotic shock from *Escherichia coli* in exponential phase. J. Biol. Chem.

